# SynPharm and the guide to pharmacology database: A toolset for conferring drug control on engineered proteins

**DOI:** 10.1002/pro.3971

**Published:** 2020-11-02

**Authors:** Jamie A. Davies

**Affiliations:** ^1^ Synthsys Centre for Systems and Synthetic Biology, Deanery of Biomedical Science University of Edinburgh Edinburgh UK

**Keywords:** biological engineering, CRISPR, drug, gene editing, pharmacological control, protein binding, protein engineering, protein structure

## Abstract

Optimizing synthetic biological systems, for example novel metabolic pathways, becomes more complicated with more protein components. One method of taming the complexity and allowing more rapid optimization is engineering external control into components. Pharmacology is essentially the science of controlling proteins using (mainly) small molecules, and a great deal of information, spread between different databases, is known about structural interactions between these ligands and their target proteins. In principle, protein engineers can use an inverse pharmacological approach to include drug response in their design, by identifying ligand‐binding domains from natural proteins that are amenable to being included in a designed protein. In this context, “amenable” means that the ligand‐binding domain is in a relatively self‐contained subsequence of the parent protein, structurally independent of the rest of the molecule so that its function should be retained in another context. The SynPharm database is a tool, built on to the Guide to Pharmacology database and connected to various structural databases, to help protein engineers identify ligand‐binding domains suitable for transfer. This article describes the tool, and illustrates its use in seeking candidate domains for transfer. It also briefly describes already‐published proof‐of‐concept studies in which the CRISPR effectors Cas9 and Cpf1 were placed separately under the control of tamoxifen and mefipristone, by including ligand‐binding domains of the Estrogen Receptor and Progesterone Receptor in modified versions of Cas9 and Cpf1. The advantages of drug control or the rival protein‐control technology of optogenetics, for different purposes and in different situations, are also briefly discussed.

## 1 INTRODUCTION

**FIGURE 1:**
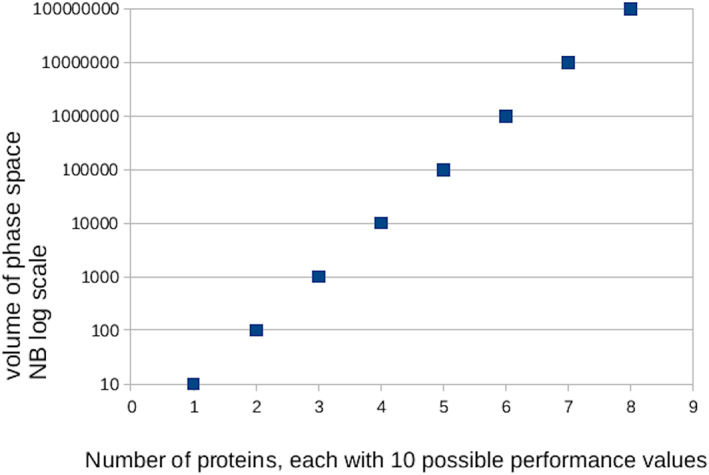
The exponential rise of the volume of phase space to be explored during the optimization of systems containing more and more engineered proteins. For simplicity, each protein is idealized to have only 10 possible performance values; the reality may well be a great deal worse

Broadly, the challenge of optimizing engineered biological systems increases exponentially with the number of components involved.^1^, ^2^, ^3^ This challenge does not lie primarily in physical construction of DNA sequences and so forth, because any difficulties here increase only linearly with system size. Rather, it arises because each extra active molecule in a system adds further dimensions to the parameter space in which the finished system will operate. To illustrate this with an example, first consider a very simple system involving only one engineered protein, the activity of which might be chosen to be any one of 10 possible values (according, for example, to the amino acid sequence of an active site). Clearly, with these restrictions, optimizing the system just requires comparing the performance of 10 possible versions. A slightly more complicated system, with two such proteins, offers 100 possible combinations of parameters. A system with three such proteins offers 1,000, and so on (Figure 1). By the time systems reach even a dozen components, the parameter space is massive and finding parameters for optimal performance is not trivial.

There are several common approaches to meet this challenge. One is mathematical modeling, which tends to be most useful when the system and its interactions with a host cell are reasonably well understood.^4^, ^5^, ^6^ Models that are mathematically tractable can be solved algebraically, while less tractable ones can be explored by computer, if necessary by the brute‐force exploration of parameter space, something that can be done much more rapidly and economically in silicon than in culture plates. Another approach, which can be used either with or as an alternative to computer modeling, is an evolutionary one of constructing many different genetic mutations of a system and selecting that with the best performance.^7^, ^8^ The genetic mutations need not be restricted to exogenous proteins; host genes may also be mutated for this “directed evolution” approach. Directed evolution is relatively straightforward if performance can be linked to fitness, so that cells harboring the best performing version of the system have a selective advantage and come to dominate the culture. Without this, though, identification and selection of cells harboring the best system, among vast numbers of other ones, becomes very difficult.

A third approach is to parameterize the system itself. Instead of being constructed with fixed components, at least parts of the system are made externally controllable, so that one physical version of the system can be used to explore different volumes of parameter space. This saves building many different versions. Most methods for doing this have centered on controlling the concentration of the protein in question by controlling the transcription of the gene that encodes it, using systems such as Tetracycline‐inducible operators (the “Tet” system^9^) or more advanced systems.^10^ These work well, and have been used for parameter exploration,^10^ but their use is restricted to controlling how much of a protein is made, not the specific activity of that protein.

An alternative approach is to engineer external regulation into the proteins themselves. Here, I describe an open‐to‐all database and tool‐set to facilitate this type of protein engineering, and describe some examples of proteins in which drug‐mediated control has been successfully engineering into DNA editing proteins.

## 2 INVERSE PHARMACOLOGY: A BACKWARDS APPROACH TO DRUGS AND TARGETS

In conventional pharmacology, a researcher begins with a target protein and attempts to find or design a small molecule that will modulate its activity in some desired way. Molecules with this property are then tested for useful kinetic properties, such as half‐life and ability to pass from vessels to tissues, and tested for safety and for efficacy. Those that pass all the tests may go on to become clinically registered drugs. The whole process is difficult, expensive, and time‐consuming; clearly it would not be an appropriate way of attaching control to new synthetic biological systems.

The conventional approach is forced on pharmacologists because they have to work with the proteins that are naturally present in the body. Designers of synthetic systems tend to engineer proteins anyway, to adapt or alter binding, enzymatic rates and so forth. This opens up the possibility of inverting the normal order of pharmacology, to begin with a drug and to design responsiveness to that drug to be a property of a new engineered protein. This approach has considerable advantages; the pharmacokinetic and safety properties of human and veterinary drugs are already well‐known and thousands are licensed for clinical use. This is the approach that the tool we describe in this article is intended to facilitate.

## 3 DESIGNING THE TOOL: WHAT DO WE KNOW, AND HOW CAN WE USE IT?

Many decades of progress in molecular pharmacology have resulted in a great deal of knowledge about which of the ~10,000 drugs and similar molecules used in clinical medicine and research bind to which of the ~3,000 human proteins that are drug targets. This knowledge is summarized in open resources, such as the IUPHAR/BPS Guide to Pharmacology database.^11^ In addition, for a significant proportion of these drugs, there is high resolution (generally crystallographic) information about precisely how the drug interacts with its target; which protein residues are involved, and how primary, secondary, and tertiary protein structures are involved in making the required protein residues available to the ligand.^12^, ^13^, ^14^, ^15^, ^16^ In principle, these datasets might be used to identify drug‐binding motifs from natural proteins that can be included in engineered proteins (by encoding them as part of the coding sequence of a transgene in the usual way).

There are, however, problems with this idea. The greatest comes from the nature of many drug‐binding sites. Proteins are complex, folded, three‐dimensional (3D) structures, and amino acids that are close to one another in space are not necessarily close to one another in the primary sequence of the peptide chain. If a drug‐binding site is formed by the spatial apposition of amino acids from many different parts of the peptide, brought to that location by the structure of the rest of the protein, it would be very difficult to re‐create the drug binding site in an engineered protein with different overall shapes and properties. The suitability of a known drug‐binding site for use in novel proteins therefore depends on the extent to which it is formed from a relatively self‐contained run of amino acids, forming a structure relatively independent of the rest of the protein. This “ligand‐binding module” can itself be highly folded, as long as it is relatively self‐contained.

Scanning drug‐protein binding structures manually to find promising examples is possible but laborious. Given that we already curate the IUPHAR/ BPS Guide to Pharmacology database, which has rich links to structural databases, we decided to build a tool to make identification of promising “drugability modules” easier.

## 4 THE SYNPHARM TOOL

The tool we have constructed, SynPharm (from “synthetic biology” and “pharmacological control”), is open to anyone to use at https://synpharm.guidetopharmacology.org/. Its home‐page presents simple statistics about the number of ligands (drugs and drug‐like molecules) and natural protein targets about which it holds binding information (at the time of writing, 515 ligands and 644 targets), search boxes, and links to tutorials and other information. It contains no new information that is not in other databases; rather, it provides new ways to interact with that information. The manner in which it was constructed and populated has been described elsewhere^17^ and this information will not be repeated here.

**FIGURE 2:**
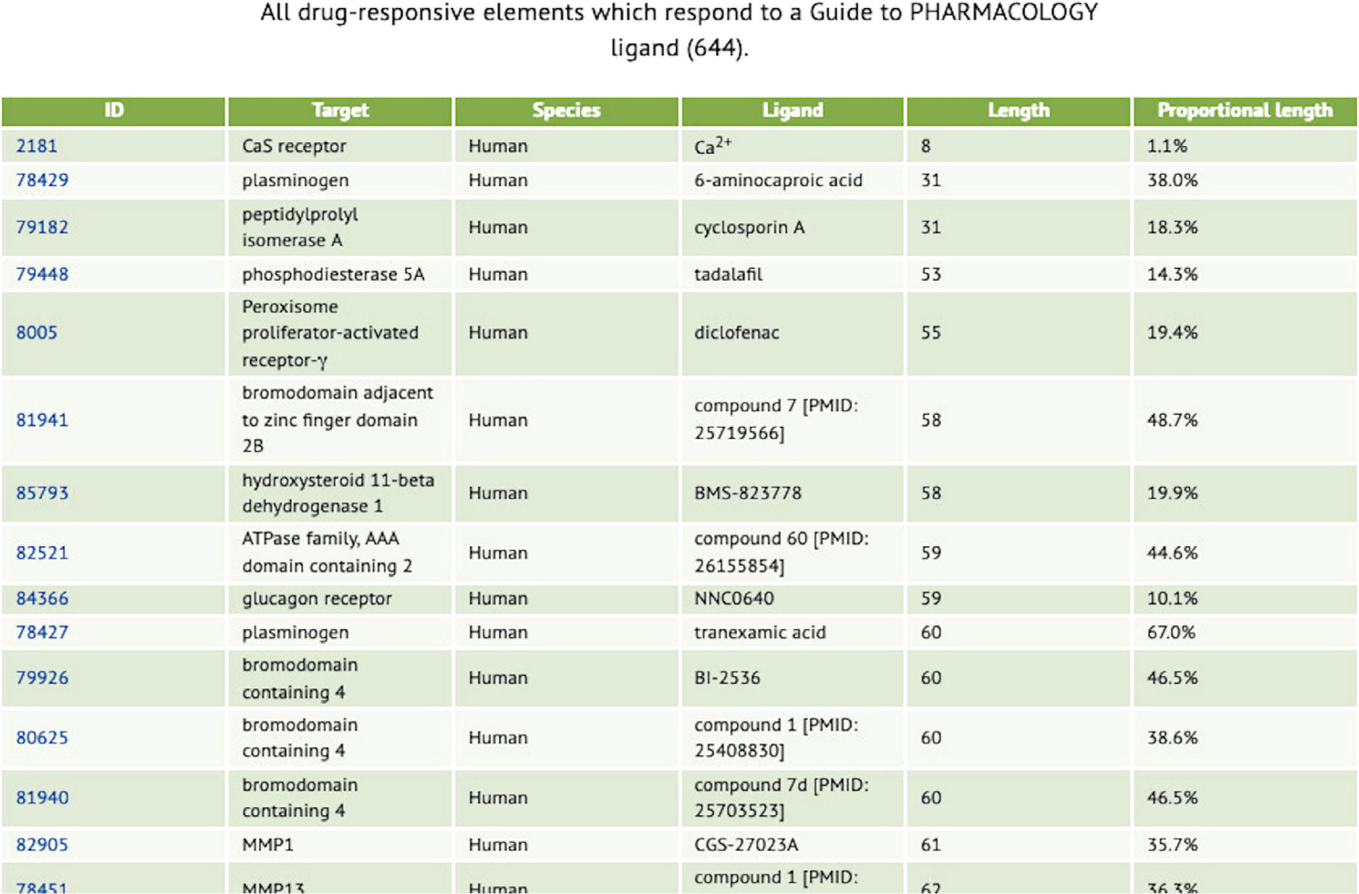
The top part of a SynPharm summary table of proteins, for which the database has structural binding data, that interact with ligands. The output has been ordered by length of ligand‐binding domain, low to high, by clicking on the “length” column header

There are several possible search strategies but the most useful to the focus of this article—identifying modules to confer drugability on engineered proteins—is simply to click “search sequences that interact with a ligand.” Doing so results in an ordered table (Figure 2), listing targets, species, ligand, length of peptide over which binding residues are scattered, and what proportion of the whole protein length this is. Clicking on any table heading will cause ordering of the table by that criterion (a second click reverses the order). Clicking on the “length” column header, as has been done in Figure 2, will order the list by the length of the binding domain, low‐to‐high. Proteins for which drug binding is a property of amino acids clustered on a short length of the peptide chain are the most likely to be useful as a source of drugability modules, but length is not the only consideration; the “independence” of that domain from the structures elsewhere in the protein is highly relevant. This independence is extremely hard to define computationally, so judgment is left to human users, who are assumed to be reasonably expert at protein engineering.

**FIGURE 3:**
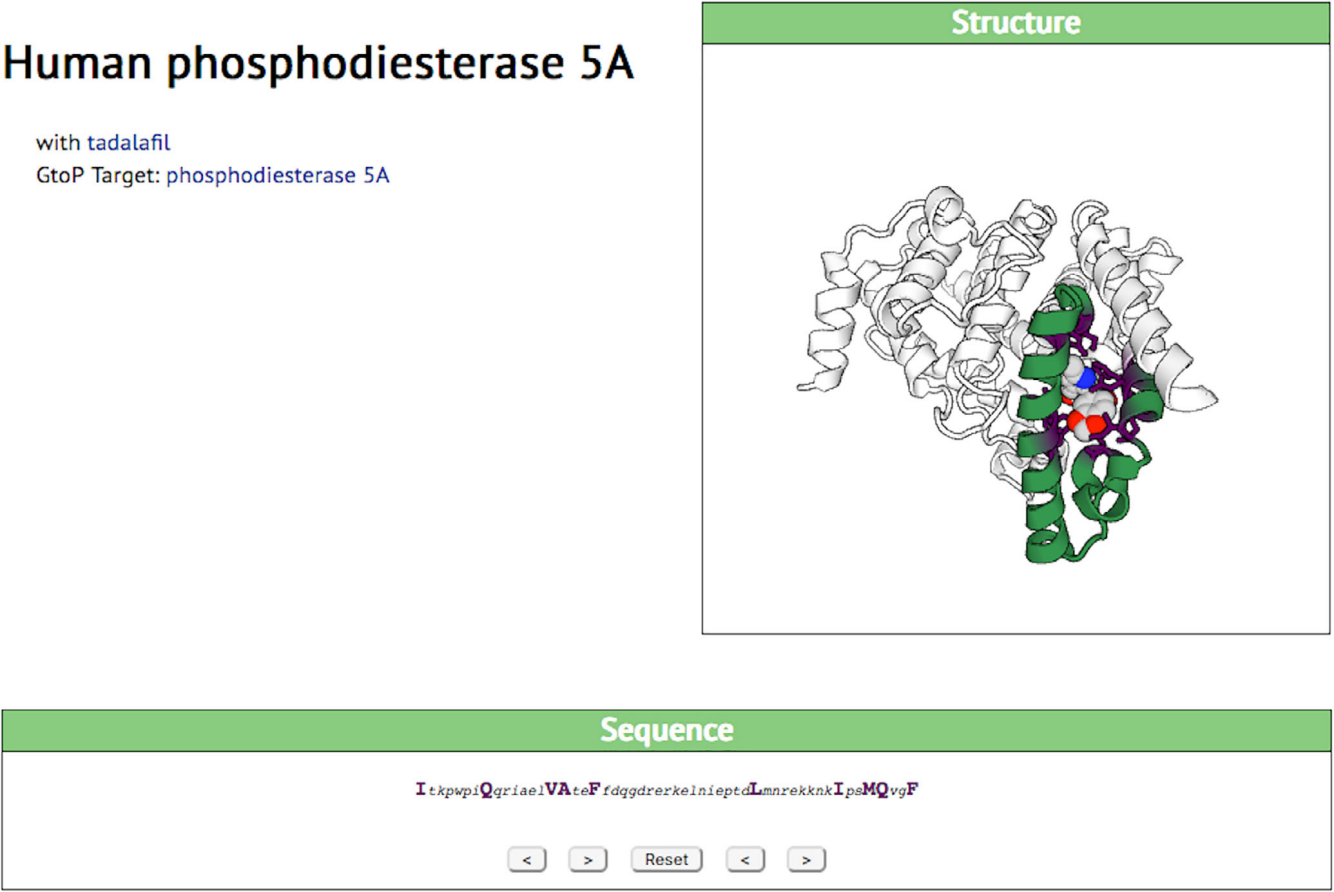
The interaction of phosphodiesterase 5A with tadalafil, as displayed on a SynPharm page. In the rotatable molecular model, the green shading represents the whole ligand‐binding sequence, also shown in single‐letter codes below. The brown, and the large amino acids in the sequence, represent amino acids directly involved in the interaction. The ligand is shown as a multi‐color chemical structure. See main text for comments on the probable (non‐) utility of this interaction for protein engineering

Clicking on the index number of any protein brings up a more detailed page. The page provides a simple display of the relevant amino acid sequence, with the ligand‐interacting amino acids highlighted to show how they cluster (or do not). It also provides a rotatable 3D model of the protein with its bound ligand, which can be used to estimate how independent of the rest of the protein is the structure of the ligand‐binding site. The interaction of the human phosphodiesterase 5A with tadalafil, for example, involves a run of only 53 amino acids but the binding structure lies at the interface of two alpha helices, the angles of which would be likely to depend strongly on details of the rest of the protein (Figure 3). This would suggest that, for ligand binding to be transferred to an engineered protein, a substantial fraction of the natural protein would need to be included (and linked to the rest of the engineered protein in a way that did not interfere with its folding). From a protein engineering point of view, this would not seem promising.

**FIGURE 4:**
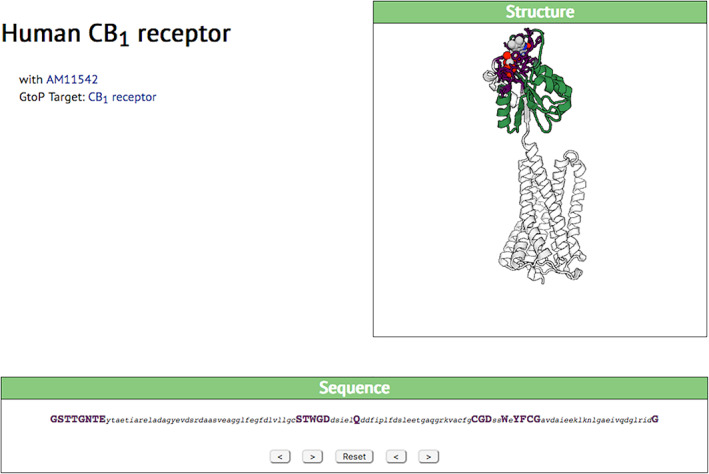
SynPharm display of the interaction of the CB1 receptor with compound AM11542. Here the interacting domain, though fairly long, is relatively independent of the rest of the molecule, linkage between the two parts of the receptor that contain many alpha helices being via a relatively flexible chain. Colors etc. are as in Figure 3

A similar view of the interaction of human CB1 receptor with the ligand AM11542, on the other hand, shows a binding site dominated by 23 amino acids widely spaced along a long sequence, but one that forms a compact and self‐contained domain of the protein that is coupled relatively flexibly to the rest (Figure 4). This suggests that this section of the natural protein might be included in an engineered protein to bring in the ligand‐binding domain.

**FIGURE 5:**
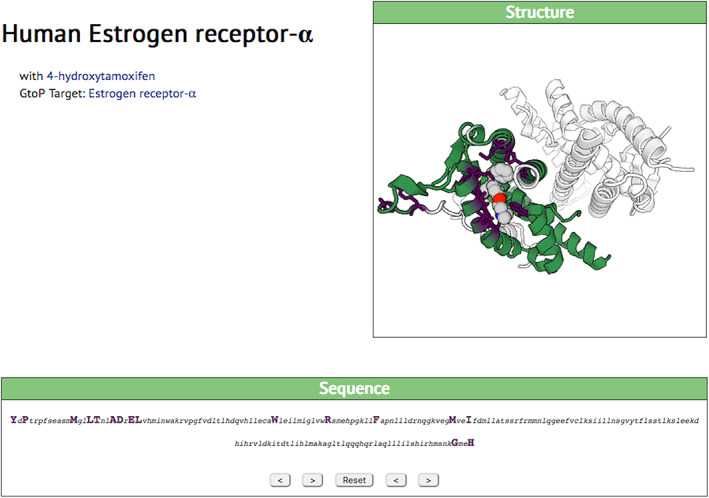
SynPharm display of interaction of Estrogen receptor α with hydroxytamoxifen, colors etc. as in Figure 3. See main text for commentary

A famous example of ligand‐interaction domains that can be ported to other, engineered proteins is provided by nuclear hormone receptors. The ligand‐binding site of ESR1 (estrogen receptor *α*), shown in Figure 5, has been connected (in a slightly mutant form) to Cre recombinase and confers tamoxifen‐dependency on that recombinase.^18^ The SynPharm tool identifies a ligand‐binding domain of 193 amino‐acids, but in fact the domain actually used to confer tamoxifen control on other molecules is around 300 amino acids long (ESR1 is 595 amino acids long in all). This acts as a warning that, though the algorithms behind SynPharm offer a useful sketch, human judgment is again needed to ensure adequate environment and “spacing” for the transferred domain in its new context.

## 5 AN EXAMPLE OF ENGINEERING DRUG CONTROL INTO EFFECTOR PROTEINS

One of the most important technologies to emerge in molecular biology has been gene editing, using the effectors of the bacterial CRISPR system (reviewed in Reference 19). These effectors can be targeted to specific genes using guide RNAs (gRNA), and can introduce either random indel‐type mutations or, in combination with templates, introduce targeted insertions or replacements.^20^ Modified versions of the effectors can also be used as transcription activators.^21^

The bacterial‐derived effectors, such as Cas9 and Cpf1, are constitutively active provided they have gRNA. For gene editing in simple two‐dimensional cultures, this is not a problem because the reagents can be introduced to cells only when editing is needed. There is, though, increasing interest in performing gene editing in solid 3D culture systems, such as organoids made from human pluripotential cells, for example, to mimic the effects of loss‐of‐heterozygosity at a locus connected with a congenital disease. In these 3D systems, access to cells for transfection with Cas9 and gRNA is highly restricted and usually only the outer layer can be reached, but transfection of cells before the organoid is made would result in gene editing happening before the developmental stage at which it is needed to mimic the disease. For these cases, having the gRNA and a drug‐controllable version of the Cas9 or Cpf1 effectors expressed in the cells all the time would allow organoids to be built in the absence of the drug, gene editing to be induced by a small molecule that can diffuse well even through an organoid.

**FIGURE 6:**
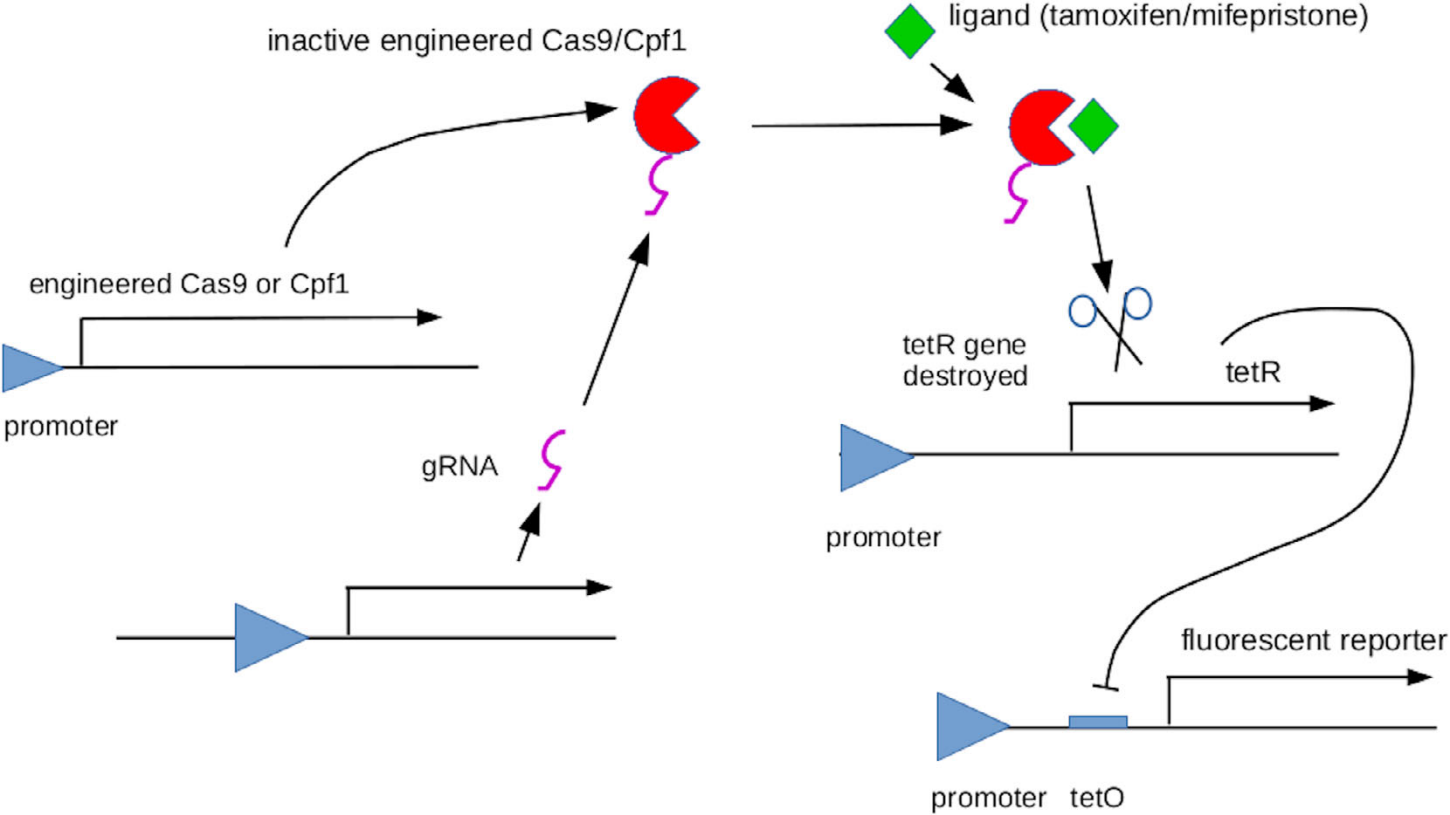
A schematic of the system used in Reference 22 to demonstrate drug control engineered separately into the Cas9 and the Cpf1 CRISPR effectors. In the absence of ligand (tamoxifen or mifepristone, depending on the ligand‐binding domain engineered into the CRISPR effector), the effector is inactive and the gene controlling TetR is intact, allowing this transcriptional repressor to act on its operator upstream of a reporter gene, to hold that gene “off.” When the ligand is present the CRISPR effector is active, the gene controlling TetR is mutated by the effector and, once residual TetR has cleared, the reporter switches “on”

We therefore engineered ligand‐binding domains from either the estrogen receptor (in the ERT2 mutant form), or the progesterone receptor, into both Cas9 and Cpf1 (Figure 6).^22^ Estrogen, progesterone and their pharmacological analogues diffuse very well in tissues because they can cross membranes. In a simple two‐dimension proof‐of‐concept study, in which gene editing destroyed a transcriptional repressor and thus freed production of a fluorescent signal from repression, the activity of these engineered CRISPR effectors was found to be highly dependent on presence of their ligands. For the tamoxifen‐inducible Cas9, for example, there was a 49‐fold difference in reporter fluorescence between dishes treated with and without 1 μM hydroxytamoxifen.^22^

## 6 CONCLUDING REMARKS

The utility of the SynPharm resource will depend on several things. One is the popularity of engineering ligand control into proteins at all. At around the same time that SynPharm was being developed, alternative technologies for protein control were maturing quickly. Optogenetic technologies, in particular, have made great advances both in control of protein expression and control of protein function, particularly that of channels.^23^ Light‐mediated control offers very high spatial and temporal resolutions offer more flexibility for control than drugs can. Indeed, this lab has begun to move toward optogenetics for these reasons.^24^, ^25^ But light has limited penetrance in deep tissues, which continues limits its utility in animal models despite recent work that has extended the depth to which light can still be used.^26^, ^27^ For engineered proteins used in this context, drug control still seems to be the best option.

The second influence on the utility of the SynPharm resource is the extent to which pre‐packaged “kit” approaches to introducing ligand control replace the need for individual protein engineers to do their own research and make their own decisions. At present, as far as the author knows, no such kits exist but if one module is developed that will work in a large range of protein hosts, then it may well dominate the field as, for example, the Tet‐operator system has come to dominate transcriptional control.^9^
